# Supplementary material for: Genome-Wide Expression Profiling Analysis of Kiwifruit GolS and RFS Genes and Identification of AcRFS4 Function in Raffinose Accumulation
Source: Int J Mol Sci. 2022 Aug 9;23(16):8836. doi: 10.3390/ijms23168836 (PMC9408211; doi:10.3390/ijms23168836)
Supplement: Supplementary file 1 [file ijms-23-08836-s001.zip › Additional file 2.pdf]

Genome-Wide Expression Profiling Analysis of Kiwifruit *GolS* and *RFS* Genes and Identification of *AcRFS4* Function in Raffinose Accumulation

Jun Yang [1,†], Chengcheng Ling [1,†], Yunyan Liu [1], Huamin Zhang [1], Quaid Hussain [2], Shiheng Lyu [2], Songhu Wang [1,*] and Yongsheng Liu [1,*]

1 College of Horticulture, Anhui Agriculture University, Hefei 350002, China; yj1904735520@sina.com (J.Y.); lingchengcheng402@163.com (C.L.); liuyunyan589@163.com (Y.L.); 17856359148@163.com (H.Z.)

2 State Key Laboratory of Subtropical Silviculture, Zhejiang A&F University, 666 Wusu Street, Hangzhou 311300, China; quaid_hussain@yahoo.com (Q.H.); lvguo2010@hotmail.com (S.L.)

* Correspondence: wangsonghu@ahau.edu.cn (S.W.); liuyongsheng1122@ahau.edu.cn (Y.L.)

† These authors contributed equally to the work.

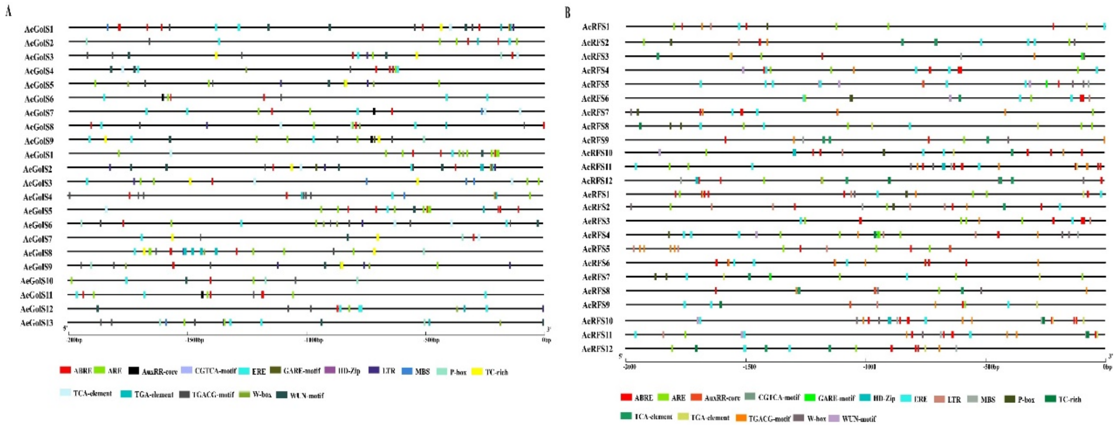

Supplementary Figure S1. Predicted cis-elements in the promoter regions of *GolS* and *RSF* genes in kiwifruit, which were presented as colored rectangular : ABA responsive element (ABRE), anaerobic responsive element (ARE), auxin responsive element (TGA-element, AuxRR-core), gibberellin responsive element (GARE-motif), ethylene responsive element (ERE, GCC-box), low-temperature-responsive element (LTR), MYB binding site (MBS), defense and stress-responsive element (TC-rich repeats), salicylic acid responsive element (TCA-element), jasmonic acid responsive element (TGACG-motif), wound-responsive element (WUN-motif), endosperm development (P-box), and WRKY binding site (W-box). The numbers on the top indicate the relative positions to the start codon. A Predicted cis-elements in the promoter regions of *GolS* genes in *A. chinensis* and *A. eriantha*; B Predicted cis-elements in the promoter regions of *RSF* genes in *A. chinensis* and *A. eriantha*.

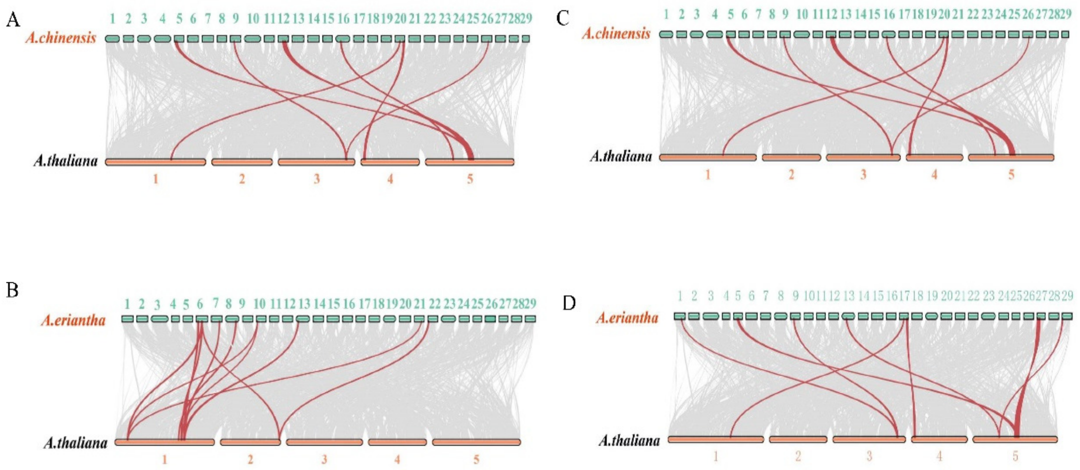

Supplementary Figure S2. Synteny analysis of *GolS* and *RFS* genes between kiwifruit and *A. thaliana*; A Synteny analysis of *AcGolS* genes between *A. chinensis* and *A. thaliana*; B Synteny analysis of *AcRFS* genes between *A. chinensis* and *A. thaliana*; C Synteny analysis of *AeGolS* genes between *A. eriantha* and *A. thaliana*; D Synteny analysis of *AeRFS* genes between *A. eriantha* and *A. thaliana*. The chromosome number is marked upon each chromosome. Gray lines in the background indicate the collinear blocks within kiwifruit and *A. thaliana*, while the red lines highlight the syntenic *GolS* and *RFS* gene pairs.

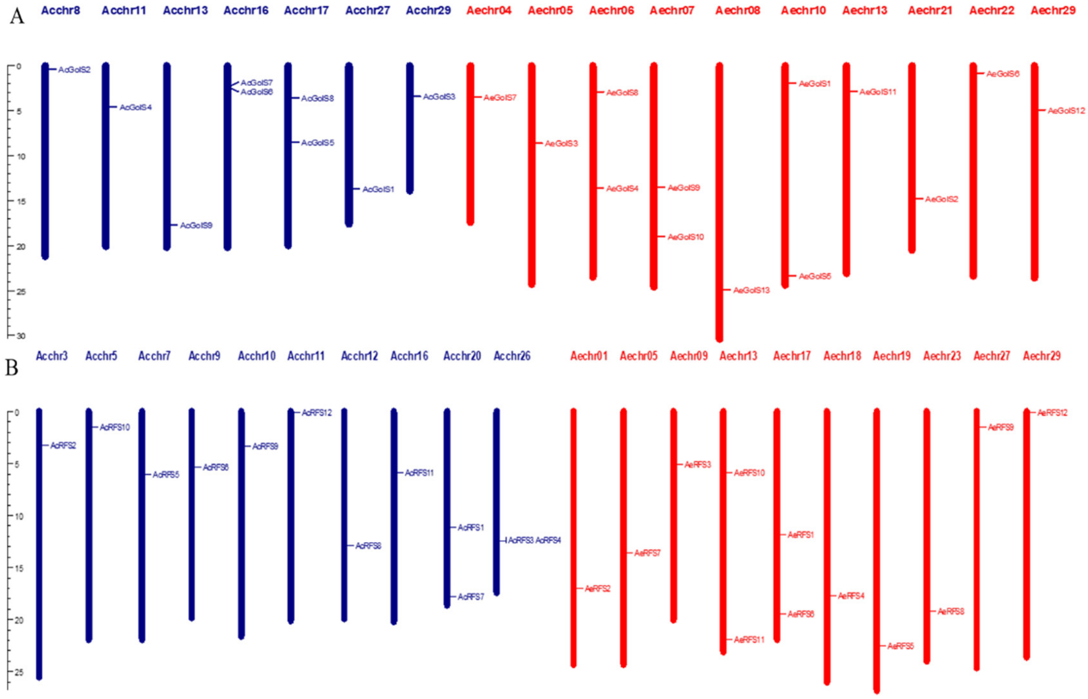

Supplementary Figure S3. Chromosomal locations of *GolS* and *RFS* genes on kiwifruit. A Chromosomal locations of *GolS* genes in *A. chinensis* and *A. eriantha*; B Chromosomal locations of *RFS* genes in *A. chinensis* and *A. eriantha*; The chromosome number is marked upon each chromosome.
